# Prognosis of young patients with monoclonal gammopathy of undetermined significance (MGUS)

**DOI:** 10.1038/s41408-021-00406-6

**Published:** 2021-02-01

**Authors:** Li Pang, S. Vincent Rajkumar, Prashant Kapoor, Francis Buadi, Angela Dispenzieri, Morie Gertz, Martha Lacy, Robert Kyle, Shaji Kumar

**Affiliations:** 1 Department of Medicine, NYC Health + Hospitals/Jacobi, Albert Einstein College of Medicine, Bronx, NY USA; 2 Division of Hematology, Department of Internal Medicine, Mayo Clinic, Rochester, MN USA

**Keywords:** Lymphoproliferative disorders, Risk factors, Myeloma, VDJ recombination, Myeloma

## Abstract

Monoclonal gammopathy of undetermined significance (MGUS) is rare in young patients (age <40 years at diagnosis), with a prevalence of <0.3%, representing ~2% of all patients with MGUS. We hypothesized that MGUS detected in young patients may be associated with a higher risk of progression. We examined 249 patients with MGUS < 40 years old. Among these, 135 patients had immune-related conditions, including infections, autoimmune and inflammatory disorders at the time of diagnosis of MGUS. The risk of progression to multiple myeloma or a related disorder at 5 years and 10 years was 6.0% and 13.8%, respectively. The size of M protein was a significant risk factor for progression (HR 4.2, 95% CI 2.2–7.9). There was a trend that the risk of progression was higher in patients without immune-related conditions (HR 2.36, 95% CI 0.85–6.52, *p* = 0.088). The M protein resolved in 36 (14%) patients, with a greater likelihood of resolution in patients with immune-related conditions (RR 1.9, 95% CI 1.02–3.6). Young patients with MGUS have a similar risk of progression as older patients, 1.4% per year. Over 50% are diagnosed in the setting of immune-related disorders. Patients with immune-related disorders may have a lower risk of progression.

## Introduction

Monoclonal gammopathy of undetermined significance (MGUS) is defined as the presence of monoclonal (M) protein ≤3 gm/dl in the peripheral blood, plasma cells <10% in the bone marrow and the absence of end organ damage or CRAB features that are related to M protein or clonal plasma cells. MGUS is perceived as a premalignant entity of multiple myeloma and lymphoplasmacytic malignancies in patients older than 50 years^1^. The progression rate of MGUS in these patients to malignant disorders is about 1% per year^2^. The prevalence of MGUS in young patients (age <40 years) is <0.3%, representing about 2% of all patients with MGUS^1,3,4^. The clinical course of young MGUS patients remains largely unknown.

A variety of risk factors have been associated with risk of progression from MGUS to other plasma cell disorders, but their impact has not been specifically evaluated in the younger patients with MGUS. Specifically, the impact of immune-related conditions, including autoimmune, infectious and inflammatory disorders occurring in the context of monoclonal gammopathy has not been studied. Lindqvist et al reported in a large case–control study that the risk of MGUS was higher in patients with all categories of immune-related conditions^5^. Similar relations were highlighted on multiple myeloma (MM) by Brown et al. that patients with immune-related conditions were 15–29% more likely to develop MM^6^. Autoimmune diseases have been associated with increase in the risk of MGUS and MM^7^. In the patients with autoimmune diseases, the age of onset of MM is earlier^8,9^. Constant stimulation to immune system in the immune-related conditions can cause B cell dysfunction and has been hypothesized as a risk for development of clonal plasma cell disorders^10,11^.

We designed the current study to better evaluate the natural history of MGUS in young patients and to specifically examine if there is a more aggressive clinical course in terms of disease progression to MM or a related disorder. Our study examined the prognosis of MGUS in a large number of young MGUS patients, and in particular the impact of concurrent immune-related conditions on the progression of MGUS.

## Patients and methods

From a prospectively maintained Dysproteinemia database at Mayo Clinic, Rochester, we identified 249 patients who were younger than 40 years at the time of diagnosis of MGUS and evaluated between January 1, 1997 and December 31, 2016. All patients met the criteria of MGUS and had a serum M protein no greater than 3 g/dl and 10% or fewer bone marrow plasma cells if a bone marrow evaluation was performed. Exclusion criteria included patients with MGUS who presented with peripheral neuropathy and subsequently diagnosed as POEMS syndrome, and those who had prior lymphoid malignancies and received chemotherapy or prolonged (>4 weeks) steroids prior to MGUS diagnosis. Patients with biclonal gammopathy were analyzed using the larger M protein. Patients with light-chain MGUS were not included in analysis involving M protein. The Mayo Clinic Institutional Review Board approved the study and it was conducted in accordance with the Declaration of Helsinki and the Health Insurance Portability and Accountability Act guidelines of 1996.

We obtained information of all patients included on age, sex, chief complaints closest to the date of MGUS diagnosis, and family history of any plasma cell disorders or hematologic malignancy. Monoclonal protein isotypes and the size of M protein were obtained from the day of MGUS diagnosis. The results of beta2 microglobulin, lactate dehydrogenase, serum free light chain ratio, bone marrow plasma cell percentage (if available) were used if the tests were done at least 3 months before progression. When M protein was detected only by immunofixation but was too small to be quantifiable on electrophoresis, the size of M protein was coded as 0.001 g/dl. Data on immune-related conditions were collected from the inpatient and outpatient records which included autoimmune, inflammatory, and infectious diseases. Patients were segregated into two groups according to the presence or absence of any of the immune-related conditions at the time of MGUS diagnosis.

The endpoint of our study was progression from MGUS to smoldering multiple myeloma (SMM), MM, macroglobulinemia, non-Hodgkin lymphoma, immunoglobulin (heavy) light chain amyloidosis or chronic lymphocytic leukemia. The patients were found to have SMM when their clinical presentations raised suspicion for disease progression and warranted further work up such as bone marrow biopsy, however, the concerning clinical symptoms were not from myeloma-defining events and the laboratory parameters did not fulfill a diagnosis of active MM. We diagnosed SMM when it met the diagnostic criteria of M spike >3 g/dl and/or 10–59% bone marrow clonal plasma cell during the follow up plus absence of myeloma-defining events. Most patients (67%) did not have bone marrow biopsy when MGUS was diagnosed consistent with our clinical practice. None of the patients in the current study received treatment at the SMM stage. We did not observe a progression to active MM in this cohort. The occurrence and date of M protein resolution were documented when immunofixation tests were negative on two consecutive assessments at least 6 months apart during the follow-up.

Chi square and Wilcoxon rank-sum tests were applied to assess the statistical significance of differences in the basic features between the group of patients with immune-related conditions and the group of patients without immune-related conditions. Progression-free survival (PFS) and overall survival (OS) for the whole cohort and between the two groups were assessed by Kaplan–Meier method, using Wilcoxon and Log-rank tests for comparison. Age, size of M protein, type of immunoglobulin, type of light chain and the presence of immune-related conditions were assessed in the Cox univariate progression model. The Cox multiparameter progression model was applied to assess the independent prognostic impact on PFS including age, the size of M protein and the presence of immune-related conditions. The above methods to analyze PFS and OS were reapplied when the patients in whom the M protein disappeared were excluded. A two-tailed *p* value < 0.05 was considered significant for all statistical tests. JMP^®^ Pro 14.0 (SAS Institute Inc., Cary, NC, USA) was used for statistical analysis.

## Results

We identified 249 patients younger than 40 years at the time of diagnosis of MGUS who met the inclusion criteria. Their baseline demographic and laboratory parameters at the time of MGUS diagnosis are shown in Table 1. The median age was 35 years; 130 (52%) were women. Most of the patients (*n* = 179, 72%) were diagnosed between the age of 30–39 years. A concurrent immune-related condition was present at the time of MGUS diagnosis in 135 (54%) patients. The immune-related conditions are shown in Table 2. A family history of hematologic malignancy was present in 14 (6%) patients, including 6 (2%) patients with plasma cell disorders. The reasons leading to the diagnosis of MGUS are reflected in the chief complaints at MGUS diagnosis, categorized by organ systems in Table 3.

**Table 1:** Characteristics of the patients.

	Entire cohort		Patients without immune-related conditions		Patients with immune-related conditions		*P* value
Characteristics	(*N* = 249)		(*N* = 114)		(*N* = 135)		
Female Sex (%)	52%		58%		47%		0.0988
Age at MGUS diagnosis	35	10–39	35	12–39	34	10–39	0.0652
median (yr)							
Size of M protein	0.27	0.001–0.43	0.36	0.001–0.60	0.20	0.001–0.30	0.0572
mean, IQR1–3 (g/dL)							
Subtypes of immunoglobulins							0.2762
IgG (no., %)	173	69.48%	82	71.93%	91	67.41%	
IgA (no., %)	25	10.04%	13	11.40%	12	8.89%	
IgM (no., %)	38	15.26%	15	13.16%	23	17.04%	
light chain only (no., %)	1	0.40%	1	0.88%	0	0.00%	
biclonal (no., %)	12	4.82%	3	2.63%	9	6.67%	
Subtypes of light chains							*0.0006
Kappa	141	59.49%	79	71.17%	62	49.21%	
Lambda	96	40.51%	32	28.83%	64	50.79%	
(biclonal excluded)							
Number of sFLC test (no., %)	69	27.71%	33	28.95%	36	26.67%	
Abnormal sFLC ratio (no., %)	12	17.39%	8	24.24%	4	11.11%	0.1506
Bone marrow plasma cell %	3.3	2–5	3.5	2–5	3.2	2–4	0.65
mean, IQR1-3 (g/dL)							
B2M, median, IQR1–3 (mg/L)	1.97	1.52–2.60	1.52	1.45–2.11	2.15	1.58–3.02	0.0835
ESR, median, IQR1–3 (mm/h)	11	4–35.5	7	2.5–30.5	12.5	5–42	0.069
LDH, median, IQR1–3 (IU/L)	183	144–243	159	142–221	197	145–253	0.0806
Family history of hematologic cancer (no., %)	14	6.00%	5	4.71%	9	7.09%	0.4485

*sFLC* serum free light chain, *IQR1-3* first to third interquartile range, *B2M* beta-2 microglobin, *ESR* erythrocyte sedimentation rate, *LDH* lactate dehydrogenase.

*P value < 0.05

**Table 2:** Immune-related conditions.

Autoimmune disorders	Inflammatory disoders	Infectious disorders
Acquired von Willebrand syndrome	Acute ischemic limb syndrome	Acute cholangitis
Ankylosing spondylitis	Alcoholic hepatitis	Bronchitis
Antiphospholipid syndrome	Angioedema	Clostridium difficile associated colitis
Asthma	Autoimmune myositis	CMV viremia
Autoimmune encephalitis	C1 esterase inhibitor deficiency	Cystitis
Autoimmune hemolytic anemia	Chronic inflammatory demyelinating polyneuropathy	Disseminated coccidiomycosis
Autoimmune hepatitis	Eosinophilic esophagitis	Disseminated herpes zoster
Autoimmune neutropenia	Glomerulonephritis	Hepatitis
Autoimmune pancreatitis	Gout arthritis	Infectious mononucleosis
Autoimmune thyroiditis	Graft-versus-host disease	Neutropenic fever
Behcet disease	Hyersensitivity reaction	Perianal abscess
Celiac disease	Hypereosinophilic syndrome	Peritonitis
Crohn’s disease	IgA nephropathy	Seasonal influenza
Cryoglobulinemia	Mastocytosis	Sinusitis
Diabetes type I	Nephrotic syndrome	
Erythema nodosum	Pleuritis	
Granulomatosis polyangitis	Post-transplant graft rejection	
Immune thrombocytopenic purpura	Seborrheic dermatitis	
Inflammatory myopathy	Seronegative spondyloarthropathy	
Leukocytoclastic vasculitis	Urticarial vasculitis	
Mixed connective tissue disease		
Multiple sclerosis		
Necrobiotic xanthogranuloma		
Necrotizing vasculitis		
Paroxysmal nocturnal hemoglobinuria		
Polyglandular autoimmune syndrome		
Primary sclerosing cholangitis		
Psoriasis		
Pyoderma gangrenosum		
Sarcoidosis		
Scleroderma		
Sjogren’s syndrome		
Systemic lupus erythematosus		
Ulcerative colitis

**Table 3:** Chief complaints for evaluations.

Category	*N*	Percentage	Category	*N*	Percentage	Category	*N*	Percentage
Neurologic	62	24.9%	Rheumatologic	26	10.4%	Constitutional	8	3.2%
Weakness/paresthesia	42	16.9%	Back pain	7	2.8%	Fever	5	2.0%
Seizures	4	1.6%	Myalgia/arthralgia	17	6.8%	Dyspnea	2	0.8%
Infarctions	1	0.4%	Others	2	0.8%	Others	1	0.4%
Dysautonomia	1	0.4%						
Dysphagia	2	0.8%	GI	24	9.6%	Infectious	7	2.8%
Dystonia	4	1.6%	Abnormal LFTs	6	2.4%	Recurrent/disseminated infections	7	2.8%
Others	8	3.2%	Abdominal pain	4	1.6%			
			Diarrhea	5	2.0%	Cardiac	5	2.0%
Nephrologic	40	16.1%	Jaundice	2	0.8%	cardiomyopathy	5	2.0%
Proteinuria	13	5.2%	Splenomegaly	5	2.0%			
AKI/ESRD	22	8.8%	Others	2	0.8%	Others	3	1.2%
Hypercalcemia	5	2.0%				N/A	8	3.2%
			Dermatologic	21	8.4%			
Hematologic	26	10.4%	Urticaria	8	3.2%			
Anemia	5	2.0%	Pyoderma gangrenaosum	3	1.2%			
Leukocytosis	1	0.4%	Urticaria pigmentosa	1	0.4%			
Leukopenia	5	2.0%	Necrobiotic xanthogranuloma	1	0.4%			
Thrombocytosis	1	0.4%	Others	8	3.2%			
Thrombocytopenia	3	1.2%						
Lymphadenopathy	2	0.8%	General evaluations	19	7.6%			
Pancytopenia	1	0.4%	General health exams	7	2.8%			
Relapsed hematologic maligancy	2	0.8%	Pretransplant evaluation	12	4.8%			
Others	6	2.4%						

The size of M protein was relatively small in our study cohort compared with that reported by others and IQR1-3 between 0.001 to 0.43 g/dl; 172 (69%) patients had small and unquantifiable M protein at diagnosis, i.e., identified on immunofixation only. The median follow-up duration for these 172 patients was 2.9 years. Only 11 (4%) patients had a serum M protein level >1.5 gm/dl. The immunoglobulin type was IgG in 69%, IgA 10%, IgM 15% and other 5% (biclonal gammopathy, *n* = 12 and light chain MGUS, *n* = 1). The type of light chain was kappa in 59% of the patients and lambda in 41%, when biclonal gammopathy was excluded. There were 69 (28%) patients who had serum free light chain (sFLC) test before progression, 33 (29%) for patients without immune-related conditions and 36 (27%) with immune-related conditions. The number of abnormal sFLC test was 8 (24%) for patients without immune-related conditions and 4 (11%) for patients with immune-related conditions (*p* = 0.20). A bone marrow examination was performed in 82 (33%) patients; the median bone marrow plasma cell percentage was 2% (IQR1-3 2–5%).

Patients without immune-related conditions tended to have a higher M protein level compared to patients with immune-related conditions (*p* = 0.057). During the follow up of 2.9 years, the M protein resolved in 36 patients. The median time to the resolution of the M protein was 1.5 years, ranging from 0.2 to 8.6 years. The median size of the M protein of these patients was not quantifiable. The M protein was more likely to resolve in patients with immune-related conditions compared to patients without immune-related conditions (RR 1.91, 95% CI 1.02–3.59; *p* = 0.03). In the 36 patients who had resolution of their initial M protein, the M protein reappeared in two patients, 6 and 9 years later, respectively.

**Fig. 1:**
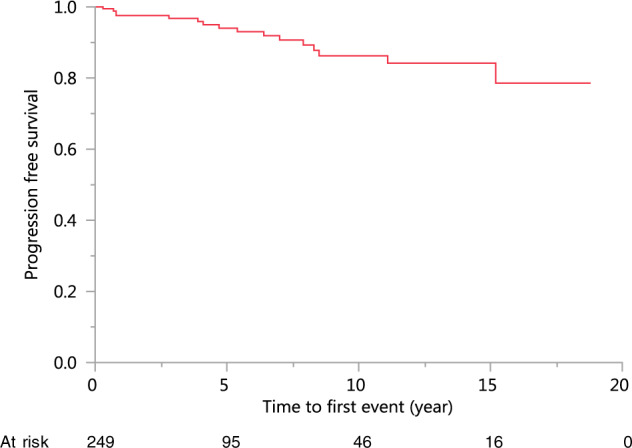
Progression-free Survival of Young Patients with MGUS. For the entire cohort, the risk of progression at 1 year, 2 years, 3 years, 4 years, 5 years and 10 years is respectively, 2.4%, 2.4%, 3.2%, 4.1%, 6.0%, and 13.8%.

Overall, 16 (6%) young MGUS patients developed progression (Table 4): 9 developed SMM, four had active multiple myeloma (MM), one had macroglobulinemia, and two had non-Hodgkin lymphoma. There were 36 patients who died and three patients who died of myeloma. The cumulative progression rate to SMM, MM or other related disorders at 5 years and 10 years was 6.0% and 13.8%, respectively (Fig. 1). In the Cox univariate analysis, the size of M protein was a significant risk factor for progression (HR 4.23, 95% CI 2.17–7.91; *p* < 0.0001). Adjusted for the presence of immune-related conditions, the size of M protein remained a strong risk factor for progression (HR 3.90, 95% CI 2.0–7.34; *p* = 0.0002). Age, type of immunoglobulin, type of light chain, and the presence of immune-related conditions did not show statistically significant impact on prognosis in the Cox model.

**Table 4:** Follow-up of the patients.

	Entire cohort		Patients without immune-related conditions		Patients with immune-related conditions		*P* value
Characteristics	(*N* = 249)		(*N* = 114)		(*N* = 135)		
Follow-up	2.9	0–18.8	1.8	0–18.8	4.2	0–18.6	*0.0332
Median, range (yr)							
Endpoint (no.)							
Any progression	16		10		6		
Smoldering multiple myeloma	9		7		2		
Multiple Myeloma	4		2		2		
Waldenstrom Macroglobulinemia	1		1		0		
Non-Hodgkin’s lymphoma	2		0		2		
Mortality	36		11		25		
Resolution of M protein	36	30.51%	10	20.00%	26	38.24%	*0.0335
(No., %)							

*P value < 0.05

**Fig. 2:**
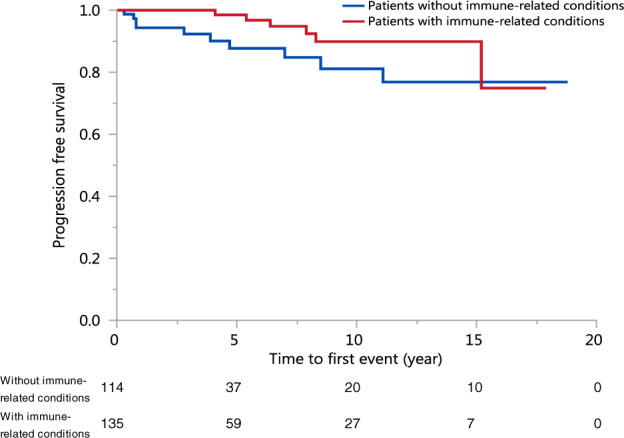
Progression-free Survival of Young Patients with MGUS with or without Immune-related Conditions. The cumulative risk of progression at 1 year, 2 years, 3 years, 4 years, 5 years, and 10 years for patients without immune-related conditions is 5.7%, 5.7%, 7.7%, 10.0%, 12.3%,and 18.9%, for patients with immune-related conditions, is 0.0%, 0.0%, 0.0%, 0.0%, 1.5%, and 10.1%. (Wilcoxon *p* = 0.0158, log-rank *p* = 0.0879).

In Fig. 2, the cumulative risk of progression at 5 years and 10 years for patients with immune-related conditions concurrently present when MGUS was first diagnosed was 1.5% and 10.1% respectively; the corresponding rate in patients without immune-related conditions at the time of diagnosis tend to be higher at 12.3% and 18.9%, respectively (HR 2.36, 95% CI 0.85–6.52, *p* = 0.088).

Similar results were seen when the patients in whom the M protein resolved (*n* = 36) were excluded. The cumulative risk of progression at 5 years and 10 years for the entire cohort was 7.6% and 17.7%, for patients with immune-related conditions was 2.2% and 14.1% and for patients without immune-related conditions was 14.0% and 21.8% (Log rank *P* = 0.084, Wilcoxon *p* = 0.032), (HR 2.49, 95% CI 0.85–7.30).

## Discussion

Our study explored the prognosis of MGUS in 249 patients younger than 40 years of age. We showed that young patients with MGUS had an average progression rate of 1.4% per year, similar to that of older patients^1,2^. It is uncommon to acquire MGUS at a young age but the early onset of MGUS did not imply a more aggressive or indolent disease process. The risk of progression to plasma cell or lymphoid disorders in young patients was predicted by the size of the serum M protein, a potent risk factor with a 3 fold higher risk of progression per unit increase. This is consistent with the previous studies in MGUS including the older patients where the size of M protein was a strong risk factor for progression^1^. The prognostic impact of the size of M protein is also evident among young MGUS patients in our study.

Our study did not find a statistically significant difference in the risk of progression between patients with immune-related conditions and patients without immune-related conditions, although there was a trend towards higher risk of progression in those without a coexisting immune disorder (HR 2.36, 95% CI 0.85–6.52, *p* = 0.088). In the early phase of follow-up, there was a higher rate of progression in patients without immune-related conditions than in the patients with immune-related conditions. In the overall long-term follow-up, this difference in progression rate disappeared.

This finding could be explained by the lead-time bias^12,13^; the survival time was artificially prolonged by early diagnosis of disease. In our study, it is likely that the patients with immune-related conditions were diagnosed earlier in the disease course of MGUS than those without immune-related conditions. They were diagnosed during the tests performed as part of clinical care for the autoimmune condition. It is possible that young MGUS patients without immune-related conditions may have acquired the potential of developing plasma cell disorder in the past^10,14–16^. In the early follow-up phase of our study, those patients without immune-related conditions had a higher rate of progression to plasma cell proliferative disorders because they had a longer history of MGUS compared with the patients with immune-related conditions who has a shorter history of MGUS. In the long-term follow up as shown in our study, there is no difference in progression free survival between the two groups of the patients.

The size of M protein in our study is relatively small compared to those in previous studies^2,5^. There are two potential explanations for this observation. First, our young MGUS patients were likely diagnosed in an earlier stage of their disease process rather than the older patients who had a longer history of undiagnosed MGUS until it is identified incidentally. The size of M protein gradually increases in the majority of patients over the disease course^17^. It is possible that the immune-related conditions is the early trigger for development of plasma cell disorders^14^, as the size of M protein was frequently reported as unquantifiable in the setting of immune-related conditions^18–20^.

Second, the unquantifiable M protein in patients with immune-related conditions might not represent bona-fide MGUS as some of them did not persist^21^. There were 36 patients in whom we observed the disappearance of M protein. In a smaller number of patients, we could not ascertain the status of the M protein as these patients were lost to follow-up after the diagnosis of MGUS. We find that the M protein was more likely to resolve in the patients with immune-related conditions compared with the patients without immune-related conditions. The patients with immune-related conditions also had a lower serum concentration of M protein. The pathophysiology behind the resolution of M protein is not clear. Murray et al reported that unquantifiable M proteins were less likely to persist as 30% of the unquantifiable M proteins disappeared in their study. They also stated among the patients with non-persistent M proteins, 55% of them had immunologic disease or received immune-modulating medication^21^. A few case series revealed similar findings that the presence of M protein was of small size and transient during acute renal failure and various infectious diseases^18–20,22,23^. The trend that patients without immune-related conditions may have higher rate of progression would be explained by the hypothesis that unquantifiable M protein does not represent the true MGUS with potential to progress. The persistence of M protein could also be interrupted by immunosuppressive medications including steroids which may reduce the serum M protein as well as cyclophosphamide which has anti-myeloma activity^24–26^.

The long-term prognosis of transient paraproteinemia is worth investigation^27^. Even though the M protein disappeared, it could recur. In the 36 patients of whom the M protein resolved, two patients were observed with the recurrence of the M protein after 6 and 9 years, respectively from its initial resolution. The type of the recurrent M protein was the same as the initial one in these two patients. Both patients were chronically on immunosuppression for their underlying diseases and the recurrence was not associated with the discontinuation of their immunosuppression. This finding is contrary to the idea that the transient paraproteinemia is a benign phenomenon. Long-term follow-up is needed to better understand this phenomenon.

There are limitations to the current study. We were not able to assess the prognostic effect of the serum free light chain ratio in our young MGUS population due to the small number of patients with available results. It is possible that the serum free light chain ratio plays a major role in prognosis as in older population but needs further study. The exact contribution of steroids to the resolution of the M protein is not known in our study as we did not have the percentage of the patients who were on steroids. It is known that steroids are one of the myeloma treatments could reduce the M protein. The follow-up duration is short in our study. Many patients who came to Mayo Clinic for complex diseases and were incidentally found to have MGUS at a young age. They lost follow up after several visits at Mayo Clinic. Possibly, their MGUS was followed up by their primary care doctor. SMM can have very protracted asymptomatic course that does not require treatment. It will provide more clinical value if future researches can include if patients require chemotherapy after they progress to SMM.

## Conclusions

Young patients with MGUS have a similar risk of progression as in older patients, 1.4% per year. Approximately 50% are diagnosed in the setting of immune-related disorders. When occurring in the setting of immune-related disorders, the M protein is smaller, more likely to resolve, and may have a lower risk of progression than in patients in whom MGUS is detected without concurrent immune-related disorder. The current study adds to our understanding of monoclonal gammopathies and their etiology.
